# Neural Computation for Rehabilitation

**DOI:** 10.1155/2014/603985

**Published:** 2014-12-28

**Authors:** Xiaoling Hu, Yiwen Wang, Ting Zhao, Aysegul Gunduz

**Affiliations:** ^1^Interdisciplinary Division of Biomedical Engineering, The Hong Kong Polytechnic University, Hung Hom, Kowloon, Hong Kong; ^2^Qiushi Academy for Advanced Studies, Zhejiang University, Zhejiang 310027, China; ^3^Howard Hughes Medical Institute, Janelia Farm Research Campus, Ashburn, VA 20147, USA; ^4^J. Crayton Pruitt Family Department of Biomedical Engineering, University of Florida, Gainesville, FL 32611, USA

With the rapid growth of ageing population, rehabilitation for neurological disorders such as Alzheimer's, Parkinson's, and stroke is one of the grand challenges faced for the coming years. Knowledge and new technologies are needed for effective rehabilitation to release the increasing demands for long-term medical treatments and healthcare, as well as help the patients regain or maintain independency in their daily lives. Successful rehabilitation depends on the understanding of the pathological mechanisms, effective methods in the treatment, and accurate evaluation of the recovery progress. Advances in neural computation provide solutions to brain modeling, quantitative neural information processing, and neural imaging. New findings in these areas also inspire novel techniques for diagnosis, rehabilitation treatments, and development of novel training devices. Considering the aforementioned trends, neural computation in the area of rehabilitation is a natural choice for the theme of this special issue. In this issue you will find thirteen high-quality, peer-reviewed articles that will provide researchers in diverse backgrounds such as engineering, neuroscience, rehabilitation, and computational sciences with the current state-of-the-art knowledge of this emerging interdisciplinary research area.

The review paper “*Neural coding for effective rehabilitation*” by the special issue editors X. Hu et al. covers a wide range of the latest breakthroughs in neural coding, neural network imaging, and neural informatics techniques with potential applications for more effective rehabilitation, as well as the advancements using electroencephalographic (EEG) and electrocorticographic (ECoG) signals in human patients for clinical applications and in intelligent robotic systems designed for interactive rehabilitation.

Rehabilitation usually is a long-term process. Its clinical success heavily depends on the accurate diagnosis and follow-up evaluations. Furthermore, for effective rehabilitation a better understanding of the relationships between physical exercise therapies and the motor outcome is essential. This special issue presents two imaging studies for enhancing diagnosis of stroke subtypes and evaluation of rehabilitation outcomes. The paper “*DWI-based neural fingerprinting technology: a preliminary study on stroke analysis*” by C. Ye et al. proposes a new methodology to identify subtypes of ischemic stroke using diffusion weighted imaging to facilitate the efficient clinical diagnosis. P. Liu et al. introduce a cost-effective ultrasonic method for poststroke muscular evaluation and monitoring the outcomes of rehabilitation training in their paper entitled “*Change of muscle architecture following body weight support treadmill training for persons after subacute stroke: evidence from ultrasonography*.”

The paper “*Gradually increased training intensity benefits rehabilitation outcome after stroke by BDNF upregulation and stress suppression*” by J. Sun et al. illustrates the effects of rehabilitation, namely, fixed training regiments versus increased training intensity on the cerebral neuroplasticity. Studying the effects of rehabilitation on the process of neuroplasticity coupled with motor outcome is extremely important for the design of therapies that are likely to expedite recovery. To this end, brain-machine interfaces (BMIs) have been a promising tool, which enables users to learn to modulate their neural activity through real-time feedback. In this issue, we highlight several articles that focus on the design of BMIs that could have significant applications for rehabilitation. The paper “*Circuit models and experimental noise measurements of micropipette amplifiers for extracellular neural recordings from live animals*” by C. H. Chen et al. presents a study on noise modeling for novel hardware designs utilizing micropipettes for extracellular recordings for BMI applications. “*Neural decoding using a parallel sequential Monte Carlo method on point processes with ensemble effect*” by K. Xu et al. introduces a new sequential Monte Carlo estimation on point processes that can accurately predict movement from neural activity in real time on GPU, providing up to 10 times faster decoding speeds compared to serial implementations. S. Ryun et al. show that it is possible to predict and delineate hand grasping from elbow flexion using ECoG signals prior to movement execution in humans. The novelty of this work is that signals utilized for the prediction were delimited to the prefrontal cortex with no input from sensorimotor areas. The next two papers focused on noninvasive recording methodologies. In “*A study on decoding models for the reconstruction of hand trajectories from the human magnetoencephalography,*” H. G. Yeom et al. present a Kalman filter approach to continuously reconstruct hand trajectories using MEG. The paper “*Nonlinear EEG decoding based on a particle filter model*” by J. Zhang et al. describes a novel nonlinear particle filter model that achieves decoding accuracies comparable to those in the literature and requires smaller training datasets. Y. Qi et al. present a BMI study for clinical applications in epilepsy in the paper “*Robust deep network with maximum correntropy criterion for seizure detection*.”

There are three papers that provide novel methodologies demonstrating promising potential applications for stroke therapy and monitoring. In “*Optogenetic activation of the excitatory neurons expressing CaMKII* *α* *in the ventral tegmental area upregulates the locomotor activity of free behaving rats*” S. Guo et al. successfully and selectively upregulated the locomotor activity of free behaving rats. The authors will further study if they can have similar results in stroke models. The paper “*A blood pressure monitoring method for stroke management*” by H. T. Ma introduces cuffless blood pressure monitors, which can be used for monitoring stroke survivors, since blood pressure is an important risk factor for stroke prognosis. H. T. Ma et al. also present a “*Muscle-based pharmacokinetic modeling of marrow perfusion for osteoporotic bone in females,*” which is a novel muscle-based pharmacokinetic modeling approach to monitor marrow perfusion.

We hope that this special issue will help to promote the further development of neural computation methodologies for rehabilitation. Improved understanding between neural plasticity and rehabilitation, as well as improved methodologies for the diagnosis of disorders and the evaluation of progress in behavior and physiology, may lead to better performance outcomes in neurorehabilitation. Such methods can also reduce the cost, duration, and overall impact of neurological disease. In addition to reducing suffering and improving quality of life, neurorehabilitation when combined with novel neural computation methods has the potential to advance our knowledge about the mechanisms of the nervous system.

